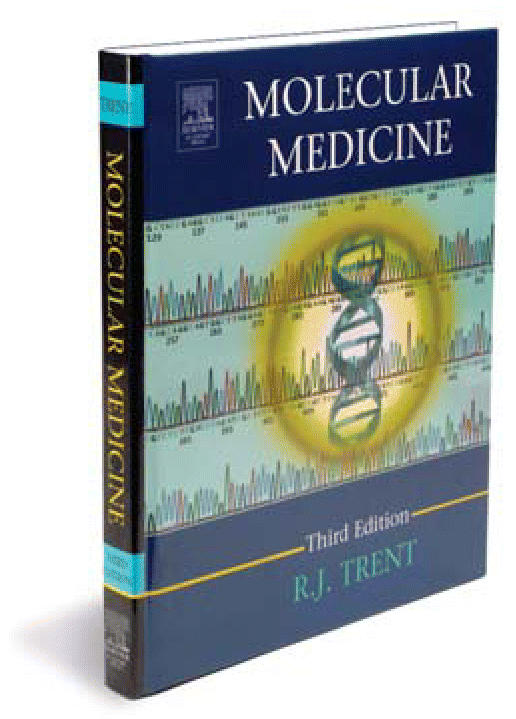# Molecular Medicine: An Introductory Text, 3rd Edition

**Published:** 2006-02

**Authors:** Y. James Kang

**Affiliations:** Y. James Kang is Professor of Medicine, Pharmacology and Toxicology, and Distinguished University Scholar at the University of Louisville, as well as a fellow of the Academy of Toxicological Sciences, editor-in-chief of Cardiovascular Toxicology, and editor of Handbook of Pharmacology and Toxicology. He is actively involved in basic and translational research in molecular medicine of heart and liver diseases.

By Ronald J. Trent

Amsterdam:Elsevier, 2005. 320 pp. ISBN: 0-12-699057-3, $79.95

The third edition of *Molecular Medicine: An Introductory Text*, like the previous editions, continues to provide a contemporary and succinct overview of DNA in medicine. Chapter topics include a history of molecular medicine, DNA, RNA, genes, and chromosomes; Mendelian genetic traits; complex genetic traits; genomics, proteomics, and bioinformatics; genetic and cellular therapies; reproduction and development; infectious diseases; forensic medicine and science; and ethical, legal, and social issues. The new chapters added to this edition represent the developments in genomics, proteomics, and bioinformatics.

Many books describe DNA in medicine, from monographs to multiple-author heavy volumes, but the uniqueness of this single-author introductory textbook of molecular medicine is that it simplifies the seemingly complex concepts of DNA in medicine so that readers can find an easy entry into the world of molecular medicine. This certainly helps medical students and biomedical researcher trainees to grasp the fundamentals of the field. This book also contains many clinical examples that make the reading more relevant to practice and raise the level of interest. Senior scientists and health care professionals will also find *Molecular Medicine* valuable because the author pulls together a comprehensive assessment, highlighting Internet-based resources that greatly assist readers in further exploring the depths and details of the related topics.

This introductory textbook presents a well-balanced incorporation of the basic concepts, applicable clinical examples, advances in molecular biology and their impact on medicine, and gaps that will be filled by further developments in DNA biology and medicine. Emphasizing the role of DNA in molecular medicine, the book provides an interesting history of the development in DNA knowledge, covering the early discovery of DNA structure to the completion of human genome project. Detailed relationships among DNA structure, biology, and medicine highlight the role of DNA in medicine, which is further enhanced by an explanation of traditional and complex genetic traits. The incorporation of genomics, proteomics, and bioinformatics adds a new dimension. The author’s presentation of the principles of using DNA information for genetic and cellular therapies and their applications for reproduction and development, infectious diseases, forensic medicine, and basic science provides a more practical view of DNA in medicine. Finally, the discussion of ethical and legal issues, of concern to the public, brings health care professionals and laboratory scientists an awareness of the social implications of molecular medicine.

The author’s definition of molecular medicine in this book—used to describe the role that knowledge of DNA is having on medical practice—may mislead readers to assume that molecular medicine has to do only with the manipulation of DNA. Posttranslational modification of proteins is a critical part of molecular medicine. Although the author has covered this aspect within the context of the book, the definition should have considered the independent role of proteins in molecular medicine as a basis for the text.

This book can be recommended to medical students, health care providers, and laboratory researchers as an entry-level textbook, introductory reference, and fundamental resource for knowledge of molecular medicine. It would easily open the door for all medical professionals to molecular medicine, its past, present, and future.

## Figures and Tables

**Figure:**